# Supporting partnerships in knowledge mobilization: what existing implementation strategies can tell us

**DOI:** 10.1186/s40900-025-00688-1

**Published:** 2025-03-12

**Authors:** Nicole E. MacKenzie, Christine T. Chambers, Kathryn A. Birnie, Isabel Jordan, Christine E. Cassidy

**Affiliations:** 1 Department of Psychology and Neuroscience, Dalhousie University, 1355 Oxford St, Halifax, B3H 4J1 NS Canada; 2 Department of Pediatrics, Dalhousie University, Halifax, NS Canada; 3 Anesthesiology, Perioperative and Pain Medicine, and Community Health Sciences, University of Calgary, Calgary, AB Canada; 4 Knowledge User and Patient Partner, Squamish, British Columbia Canada; 5 School of Nursing, Dalhousie University, Halifax, NS Canada

**Keywords:** Implementation science, Partnerships, Integrated knowledge translation

## Abstract

**Background:**

The need for partnership between knowledge producers and knowledge users to foster effective implementation is well-established in the implementation science literature. While many theories, models, and frameworks (TMF) have been developed to guide knowledge mobilization (KM) activities, seldom do these frameworks inform approaches for establishing and maintaining KM partnerships (i.e., relationships between researchers and individuals with relevant expertise in KM activities). Thus, there is a significant knowledge-to-action gap related to operationalizing engagement in partnerships and leveraging the evidence that exists to support them. Given the abundance of TMFs, it is prudent to consider whether any may be suitable to inform approaches to partnership. The aim of this commentary is to discuss the necessity for strategies to support engagement in partnerships for KM activities, as well as to explore the potential to apply strategies from an existing implementation taxonomy to inform partnerships approaches in KM.

**Main body:**

Using a case study, this commentary explores the opportunity to apply existing implementation strategies put forward by the Expert Recommendations for Implementing Change (ERIC) taxonomy to inform partnership strategies. This case study utilized qualitative evidence from a qualitative study about KM in children’s pain management informed by the Consolidated Framework for Implementation Research (CFIR). It explored partner perspectives (i.e., knowledge producers and users) on factors that supported their engagement in KM activities. The factors generated were subsequently mapped onto the ERIC taxonomy to identify relevant strategies to support partnerships development for KM activities (e.g., *shared goals among team members* mapped onto the ERIC strategy *Build a Coalition*). Each factor generated was determined to have a corresponding ERIC strategy to support the operationalization of that factor.

**Conclusions:**

This case example and discussion bolster the utility of existing taxonomies and frameworks to support the development and sustainability of partnerships to support engagement in KM activities, a promising next step for developing strategies to support partnerships. Opportunities for future development are also discussed, including identifying other theories, models, and frameworks that may contribute to a comprehensive suite of empirically informed partnership strategies, as well as the necessity to make strategies and approaches available to non-specialist audiences.

## Abstract (plain language summary)

When it comes to generating and spreading knowledge about health research, it is critical for researchers to do this in partnership with patients, caregivers, health professionals and others who may be impacted by, or benefit from, this knowledge. This is especially important in children’s pain, where the sharing and application of knowledge has historically been challenging and slow. While partnership is becoming more recognized as an important research and knowledge sharing practice, the frameworks available in implementation science do not typically describe how to engage in partnerships. The purpose of this commentary was to discuss the importance of using strategies to support how partners are included in knowledge sharing activities. It also aimed to explore whether strategies from an existing implementation framework could be used to support partnership between researchers, patients, caregivers, and health professionals. The authors present a case study developed from a previous research study that explored the perspectives of these different partners on what supported their engagement in KM activities. Review of these qualitative results generated 16 factors that were related to supporting partnership which were mapped on to the Expert Recommendations for Implementing Change (ERIC) taxonomy. Each factor had a corresponding ERIC strategy. This means that the ERIC strategies could be used to inform partnership approaches. This work suggests that existing implementation science frameworks may be useful to inform partnership approaches. The commentary also highlights opportunities to make frameworks more practical for non-academic partners, like patient partners, to support their engagement in partnerships.

## Background

Engagement and partnership with knowledge users (i.e., individuals who can utilize knowledge to inform clinical and policy decision making) are the cornerstones of knowledge co-production approaches (i.e., collaborative and participatory approaches to research) [1]. These approaches, such as integrated knowledge translation (IKT), leverage the lived expertise of those who may use or be impacted by evidence to inform how it is generated, disseminated, and applied [2]. Partnership with knowledge users, such as patients, health professionals, and researchers, ensures the relevance of the evidence generated, increases the uptake of evidence in practice and policy decisions, supports the spread and scale of evidence-based interventions, and ultimately improves health service outcomes [2–4]. While partnership often occurs in the context of knowledge co-production (i.e., research), the inclusion of knowledge user perspectives is also crucial to knowledge mobilization (KM; i.e., the dissemination, implementation, synthesis, and exchange of evidence) activities [5], to ensure their relevance and impact, making partnership a key component of KM. There are many theories, models, and frameworks (TMFs) within the field of implementation science available to inform strategies or approaches to evidence dissemination and implementation [6]. Few of these, however, provide an evidence-based approach to the implementation of partnerships for knowledge co-production or KM activities, and typically identify partnership as a strategy without recommendations on how to enact it [7]. This means that there is limited guidance to inform how partnerships are developed and maintained throughout the duration of a KM initiative and beyond. Filling this gap is essential to support effective partnership in KM activities; however, with the abundance of TMFs available, it is prudent to explore the opportunity to utilize existing TMFs to inform these activities so as to potentially avoid adding to what is already a complex literature. As such, it is worthwhile exploring the presence of relevant strategies within existing TMFs to explore whether such strategies may inform partnership activities within KM. With this in mind, the purpose of this commentary is twofold. First, it aims to provide background on the necessity to identify specific strategies to inform how individuals are engaged as partners within KM activities. Second, this paper aims to explore the potential opportunity to use strategies in existing TMFs to inform partnership activities. This is explored using a brief case example, where factors from a previous qualitative study on partnership in KM are mapped onto the strategies from one taxonomy (i.e., ERIC taxonomy) to determine the potential for these strategies to inform partnership activities. These aims are followed by a discussion around the opportunity to use existing TMFs to inform partnership activities in KM.

## Main body

### The issue: a growing gap between implementation science and partnership practices in KM

A goal of implementation science is to inform the practice of implementation and other KM activities to close the knowledge-to-action gap [8]. There is, however, a growing gap between the science and practice of implementation [9, 10], with a specific issue being the lack of evidence available to inform strategies for engaging in partnerships for KM activities [7]. There are several TMFs and co-production approaches, such as IKT, that inform how evidence is generated and mobilized. There is also an abundance of implementation science TMFs. It is often unclear which, if any, are appropriate to inform partnership approaches, and the processes proposed may not always align with the context or purpose of the partnership [11]. Moreover, most lack specific guidance around how to operationalize engagement in partnerships [12, 13]. Beyond a lack of practical strategies for partnership, TMFs themselves are typically developed with implementation specialists and scholars in mind, and often without patient partners. Moreover, there is insufficient guidance to inform how strategies are tailored to different contexts, or how best to engage different partners, which may lead to poor applicability among knowledge users in practice [9, 14]. For patient partners in particular, this creates a risk for tokenization or exclusion from implementation science. As the emphasis on patient partnership has increased over time, so too have efforts to create frameworks that inform partnership practices. For example, the Patient Engagement in Research Framework and the Strategy for Patient Oriented Research offer guiding principles to consider when engaging in partnerships [15, 16]. There is also a growing literature on key partnership principles, including meaningful engagement, relationship development, balancing power dynamics, and reciprocity [17, 18]. While building awareness of these principles is critical, and research has identified some strategies to support partnership (e.g., location choices, employing connectors) [17], many of these frameworks do not offer applied strategies to inform how partnership is carried out, and especially not in the context of KM. As these shortcomings are recognized, a key goal of the field has been to fill these gaps and identify strategies to support partnership between knowledge producers and users [11, 19]. 

### A potential solution: addressing the implementation science and KM gap with existing theories, models, and frameworks

While the use of implementation science TMFs to develop and maintain partnerships has been limited, they may be an important resource to inform the development of, and engagement in, partnerships, including to support KM practice. TMFs can provide structure to partnerships, from identifying relevant knowledge users, informing when to engage partners, and assigning roles [20]. Some TMFs have also been connected to specific implementation strategies, such as the Expert Recommendations for Implementing Change (ERIC) taxonomy, which can support the transfer of evidence into practice [21, 22]. Just as implementation strategies have been developed based on existing research and TMFs, strategies for partnership may also be developed in a similar way. Evidence for utilizing existing TMFs or strategies for implementation to inform partnerships could reduce research waste by tailoring existing approaches to the KM context while also reducing the need for research to develop new strategies. In doing so, the development or selection of strategies for partnership should be directly informed by the needs of all partners involved – a process that could be informed by any relevant implementation science TMF [20]. 

The translation of TMFs to specific implementation strategies has been explored within the context of some existing frameworks. The ERIC taxonomy provides specific strategies to address the domains captured within the Consolidated Framework for Implementation Research (CFIR; i.e., innovation, outer setting, inner setting, individuals, implementation process), providing opportunity to address these factors in practice [21–23]. The ERIC taxonomy not only provides discrete strategies to address implementation goals, but also defines and describes the potential applications of these strategies [21]. While the strategies in the ERIC are intended to inform implementation practice, a question that arises is whether there are opportunities to tailor such taxonomies or other TMFs to inform efforts to engage in partnership.

### Case example: applying the ERIC taxonomy to inform partnership strategies

Partnership and KM have become priorities in children’s pain management, given the longstanding knowledge to action gap between pain management strategies and practice, attributed, in part, to lack of knowledge of evidence and a need for greater support with partnership and implementation [24]. To illustrate the potential application of an existing implementation science TMF to inform partnership strategies (i.e., the ERIC taxonomy), findings from a previously published research study by MacKenzie and colleagues [25] were used to develop a case example. These findings that constitute the case helped to illustrate the utility of the ERIC taxonomy to inform a potential partnership approach for KM within children’s pain. The purpose of this previously published qualitative study was to understand the perspectives of different partner types (i.e., patients/caregivers, health professionals, and researchers) when it comes to supporting their engagement in KM activities within children’s pain.

#### Approach

The study’s interview guide was informed by the CFIR, where each domain was explored through semi-structured questions to understand what impacted partners’ engagement in previous KM activities within pediatric pain. Subsequent reflexive thematic analysis considered the relevance of these domains when presenting data on areas of importance to support engagement in KM. The themes generated from this study pertained to four organizing concepts: team dynamics, role of leadership, policy influence, and social influence. Participants spoke to facilitators of KM that in their experiences, supported their ability to engage in KM activities within a partnership. From these data, 16 factors were generated that spoke to the facilitators and considerations generated for effective partnership and engagement in KM [25]. Each of the four organizing concepts were represented among the factors generated, with greater representation from the organizing concepts that were more closely related to partnership. There were 7 factors initially derived from the role leadership organizing concept (i.e., receiving routine project updates, flexibility in how communication/meetings take place, buy in from team members, collaborative leadership style, structured opportunities to participate in KM activities; fit of the KM initiative and plan with the intended context, flexibility in the implementation plan), 6 factors from team dynamics (i.e., teams with diverse expertise, having knowledge of the evidence to be shared/applied, freedom to share perspectives openly with team members, shared goals among team members, presence of a “champion” for a KM initiative, having knowledge of how to do KM), 2 factors from social influence (i.e., reputation of the individual/organization whose initiative is being implemented, maintaining engagement with peer/professional networks), and 1 factor from policy influence (i.e., resources from institutions/decision makers to engage in KM).

While interpreting these findings, a question arose as to whether there were specific strategies that could be developed to support partnership for KM. To address this unique question, the 16 factors were mapped on to corresponding strategies presented in the ERIC taxonomy for this new paper. The goal of this mapping process was to identify relevant strategies that could support the implementation of partnerships within a KM context. The ERIC taxonomy was selected given the relevance of the strategies to the CFIR, which underpinned the previous study. Each ERIC strategy was reviewed by one reviewer (NEM) and those which raised similar concepts, definitions, and/or examples were determined to have correspondence to the factors generated from the qualitative data. ERIC strategies and partnership factors that were determined to correspond were then reviewed by a second reviewer (CEC), who was a co-investigator in the preceding qualitative study. Each connected strategy and factor were discussed and then either agreed upon or further discussed and resolved through consensus. During the consensus discussion, both reviewers discussed whether there was an example where the strategy could in fact inform the partnership need. Consensus was reached when a partnership factor was either determined to be appropriate to remain paired with a strategy or factors were unpaired with their initial strategy and re-paired with another where appropriate.

#### Mapping outcomes

Upon completing this process, each of the 16 factors were determined to have at least one corresponding ERIC strategy that could potentially inform how it could be facilitated and implemented, with a total of 37 ERIC factors identified (see Table 1 for the mapping outcomes). The corresponding cluster for each ERIC strategy, as found within Waltz and colleagues work [22], was also presented. All nine clusters were represented in the results, with the most common clusters being “develop stakeholder interrelationships” which occurred nine times, and “train and educate stakeholders” which occurred six times.

**Table 1:** Mapping KT engagement factors to ERIC strategies

KT engagement factors	Factor definition	ERIC cluster	ERIC strategy	Strategy definition
1. Teams with diverse expertise	Teams that consist of individuals with different professional backgrounds and experiences interacting with the healthcare system.	Engage consumers	Intervene with patients/consumers to enhance uptake and adherence	Develop strategies with patients to encourage and problem solve around adherence
			Involve patients/consumers and family members	Engage or include patients/consumers and families in the implementation effort
			Prepare patients/consumers to be active participants	Prepare patients/consumers to be active in their care, to ask questions, and specifically to inquire about care guidelines, the evidence behind clinical decisions, or about available evidence-supported treatments
		Use evaluative and iterative strategies	Obtain and use patients/consumers and family feedback	Develop strategies to increase patient/ consumer and family feedback on the implementation effort
		Provide interactive assistance	Provide local technical assistance	Develop and use a system to deliver technical assistance focused on implementation issues using local personnel
		Train and educate stakeholders	Provide ongoing consultation	Provide ongoing consultation with one or more experts in the strategies used to support implementing the innovation
		Develop stakeholder interrelationships	Use advisory boards and workgroups	Create and engage a formal group of multiple kinds of stakeholders to provide input and advice on implementation efforts and to elicit recommendations for improvements
2. Having knowledge of the evidence to be shared/applied	Knowledge of the intervention, scientific findings, or other factors related to the change to be made through the KM initiative.	Train and educate stakeholders	Conduct educational meetings	Hold meetings targeted toward different stakeholder groups (e.g., providers, administrators, other organizational stakeholders, and community, patient/consumer, and family stakeholders) to teach them about the clinical innovation
			Conduct educational outreach visits	Have a trained person meet with providers in their practice settings to educate providers about the clinical innovation with the intent of changing the provider’s practice
			Develop educational materials	Develop and format manuals, toolkits, and other supporting materials in ways that make it easier for stakeholders to learn about the innovation and for clinicians to learn how to deliver the clinical innovation
		Provide interactive assistance	Provide clinical supervision	Provide clinicians with ongoing supervision focusing on the innovation. Provide training for clinical supervisors who will supervise clinicians who provide the innovation
3. Fit of the KM initiative and plan within the intended context	How well the KM initiative fits within the context it will be shared in or applied, and how well the plan considers characteristics such as resources available, staffing/personnel, workflow, demands on staff, etc.	Adapt and tailor to context	Promote adaptability	Identify the ways a clinical innovation can be tailored to meet local needs and clarify which elements of the innovation must be maintained to preserve fidelity
			Tailor strategies	Tailor the implementation strategies to address barriers and leverage facilitators that were identified through earlier data collection
		Change infrastructure	Change physical structure and equipment	Evaluate current configurations and adapt, as needed, the physical structure and/or equipment (e.g., changing the layout of a room, adding equipment) to best accommodate the targeted innovation
		Support clinicians	Revise professional roles	Shift and revise roles among professionals who provide care, and redesign job characteristics
4. Reputation of the individual/organization whose initiative is being implemented.	Whether the KM initiative was developed and/or associated with someone well-known and influential in one’s field of research/practice.	Develop stakeholder interrelationships	Inform local opinion leaders	Inform providers identified by colleagues as opinion leaders or “educationally influential” about the clinical innovation in the hopes that they will influence colleagues to adopt it
5. Maintaining engagement with peer/professional networks	Connectedness with a network of individuals with relevant interests, professional backgrounds, expertise, etc.	Develop stakeholder interrelationships	Promote network weaving	Identify and build on existing high-quality working relationships and networks within and outside the organization, organizational units, teams, etc. to promote information sharing, collaborative problem-solving, and a shared vision/goal related to implementing the innovation
			Capture and share local knowledge	Capture local knowledge from implementation sites on how implementers and clinicians made something work in their setting and then share it with other sites
		Support clinicians	Develop resource sharing agreements	Develop partnerships with organizations that have resources needed to implement the innovation
6. Receiving routine project updates	Receiving updates on a regular basis regarding a KM project as it evolves, new steps are planned, or issues arise.	Engage consumers	Involve patients/consumers and family members	Engage or include patients/consumers and families in the implementation effort
7. Flexibility in the implementation plan	A plan for how the KM initiative will proceed that outlines a general road map but leaves opportunities to change/adjustment as needed.	Use evaluative and iterative strategies	Develop a formal implementation blueprint	Develop a formal implementation blueprint that includes all goals and strategies. The blueprint should include the following: (1) aim/purpose of the implementation; (2) scope of the change (e.g., what organizational units are affected); (3) timeframe and milestones; and (4) appropriate performance/progress measures. Use and update this plan to guide the implementation effort over time
		Develop stakeholder interrelationships	Obtain formal commitments	Obtain written commitments from key partners that state what they will do to implement the innovation
		Adapt and tailor to context	Promote adaptability	Identify the ways a clinical innovation can be tailored to meet local needs and clarify which elements of the innovation must be maintained to preserve fidelity
		Use evaluative and iterative strategies	Purposely re-examine the implementation	Monitor progress and adjust clinical practices and implementation strategies to continuously improve the quality of care
		Support clinicians	Revise professional roles	Shift and revise roles among professionals who provide care, and redesign job characteristics
8. Flexibility in how communication/meetings take place	Options for a range of methods to engage in communication and meetings, such as virtual meetings, email updates, brief one-on-one updates as needed, etc.	Develop stakeholder interrelationships	Organize clinician implementation team meetings	Develop and support teams of clinicians who are implementing the innovation and give them protected time to reflect on the implementation effort, share lessons learned, and support one another’s learning
9. Freedom to share perspectives openly with team members	The context creates a sense of safety and trust in which individuals can share their perspectives and make contributes openly.	Develop stakeholder interrelationships	Build a coalition	Recruit and cultivate relationships with partners in the implementation effort
		Use evaluative and iterative strategies	Obtain and use patients/consumers and family feedback	Develop strategies to increase patient/consumer and family feedback on the implementation effort
		Provide interactive assistance	Facilitation	A process of interactive problem solving and support that occurs in a context of a recognized need for improvement and a supportive interpersonal relationship
10. Buy-in from team members	Individuals’ investment and interest in participating in the KM activity.	Utilize financial strategies	Alter incentive/allowance structures	Work to incentivize the adoption and implementation of the clinical innovation
		Provide interactive assistance	Facilitation	A process of interactive problem solving and support that occurs in a context of a recognized need for improvement and a supportive interpersonal relationship
		Develop stakeholder interrelationships	Identify early adopters	Identify early adopters at the local site to learn from their experiences with the practice innovation
11. Shared goals among team members	Team members all work toward a common goal	Develop stakeholder interrelationships	Build a coalition	Recruit and cultivate relationships with partners in the implementation effort
			Conduct local consensus discussions	Include local providers and other stakeholders in discussions that address whether the chosen problem is important and whether the clinical innovation to address it is appropriate
12. Collaborative leadership style	Leadership that takes all team member perspectives and opinions into consideration and shares decision making opportunities with others.	Train and educate stakeholders	Create a learning collaborative	Facilitate the formation of groups of providers or provider organizations and foster a collaborative learning environment to improve implementation of the clinical innovation
		Provide interactive assistance	Facilitation	A process of interactive problem solving and support that occurs in a context of a recognized need for improvement and a supportive interpersonal relationship
13. Structured opportunities to participate in KM activities	Opportunities where individuals are intentionally given time and space to share their opinions during meetings, contributing to developing ideas, outputs, etc.	Engage consumers	Involve patients/consumers and family members	Engage or include patients/consumers and families in the implementation effort
		Use evaluative and iterative strategies	Obtain and use patients/consumers and family feedback	Develop strategies to increase patient/consumer and family feedback on the implementation effort
14. Presence of a “champion” for a KM initiative	An individual who empowers, promotes, and motivates other team members working on a KM initiative.	Develop stakeholder interrelationships	Identify and prepare champions	Identify and prepare individuals who dedicate themselves to supporting, marketing, and driving through an implementation, overcoming indifference or resistance that the intervention may provoke in an organization
15. Resources from institutions/decision makers to engage in KM	Availability of resources (e.g., time, funding, personnel, etc.) to make carrying out KM initiatives feasible.	Utilize financial strategies	Access new funding	Access new or existing money to facilitate the implementation
		Develop stakeholder interrelationships	Develop academic partnerships	Partner with a university or academic unit for the purposes of shared training and bringing research skills to an implementation project
			Involve executive boards	Involve existing governing structures (e.g., boards of directors, medical staff boards of governance) in the implementation effort, including the review of data on implementation processes
		Train and educate stakeholders	Work with educational institutions	Encourage educational institutions to train clinicians in the innovation
16. Having knowledge of how to do KM	Skills, awareness of processes, relevant theories/frameworks, and other factors related to carrying out or participating in a KM initiative.	Train and educate stakeholders	Conduct educational meetings	Hold meetings targeted toward different stakeholder groups (e.g., providers, administrators, other organizational stakeholders, and community, patient/consumer, and family stakeholders) to teach them about the clinical innovation
			Conduct educational outreach visits	Have a trained person meet with providers in their practice settings to educate providers about the clinical innovation with the intent of changing the provider’s practice
			Develop educational materials	Develop and format manuals, toolkits, and other supporting materials in ways that make it easier for stakeholders to learn about the innovation and for clinicians to learn how to deliver the clinical innovation
			Provide ongoing consultation	Provide ongoing consultation with one or more experts in the strategies used to support implementing the innovation
			Shadow other experts	Provide ways for key individuals to directly observe experienced people engage with or use the targeted practice change/innovation
			Use train-the-trainer strategies	Train designated clinicians or organizations to train others in the clinical innovation
		Develop stakeholder interrelationships	Recruit, designate, and train for leadership	Recruit, designate, and train leaders for the change effort
			Use an implementation advisor	Seek guidance from experts in implementation
			Visit other sites	Visit sites where a similar implementation effort has been considered successful
		Provide interactive assistance	Provide local technical assistance	Develop and use a system to deliver technical assistance focused on implementation issues using local personnel

**Multiple ERIC Strategies Can Inform a Single Partnership Factor.** In some instances, there were multiple ERIC strategies that could potentially inform the implementation of a single partnership factor. The number of ERIC strategies assigned to each factor ranged from one to ten (Mode = 3). For example, the partnership factor *shared goals among team members* was mapped onto the ERIC strategy *Build a Coalition*, defined as “recruit and cultivate relationships with partners in the implementation effort,” as well as the ERIC strategy *Conduct Local Consensus Discussions*, defined as “include local providers and other stakeholders in discussions that address whether the chosen problem is important and whether the clinical innovation to address it is appropriate.” In this example, both ERIC strategies provide concrete actions to enact the aim of team members working toward a common goal, and specifically suggests facilitating this by recruiting to develop partnerships as well as leading meetings to discuss whether the goal is relevant to those partners who have been recruited.

**Single ERIC Strategies Can Inform Multiple Partnership Factors**. In other instances, multiple ERIC strategies were determined to be relevant to multiple partnership-related factors. For example, the ERIC strategy *Obtain and Use Patients/Consumers and Family Feedback*, defined as “develop strategies to increase patient/consumer and family feedback on the implementation effort,” was determined to be relevant to support the partnership factors *teams with diverse expertise*, freedom *to share perspectives openly with team members*, and *structured opportunities to participate in KM activities*. The opportunity to apply an individual strategy to address multiple factors of relevance within a context speaks to the flexibility and utility of these strategies to meet multiple needs through fewer efforts. That is, the opportunity for a single ERIC strategy to address multiple partnership needs speaks to the efficiency of using an existing framework, as implementing fewer strategies may result in less time and resources needed to engage in partnership and KM effectively, while addressing multiple aims or goals. This in turn may improve the feasibility of using existing implementation science TMFs to inform partnership, which may lead to improved partnership experiences and KM outcomes.

**Opportunities to Tailor ERIC Strategies**. In other instances, ERIC strategies were determined to be related to partnership factors, though it would be necessary to tailor them to be more relevant to the KM contexts or those being engaged in a partnership. For example, the partnership factor *flexibility in how communication/meetings take place* was mapped on to the ERIC strategy *Organize Clinician Implementation Team Meetings*, defined as “develop and support teams of clinicians who are implementing the innovation and give them protected time to reflect on the implementation effort, share lessons learned, and support one another’s learning.” While this strategy addresses how to accommodate one type of knowledge user’s (i.e., clinicians) needs concerning flexibility, it does not specifically address how to tailor to other types of knowledge users’ specific needs (e.g., patients and caregivers). This ERIC strategy still provides a helpful approach to accommodate clinicians but would need to be tailored to address the needs of other knowledge users, such as patients and caregivers who often have competing personal and health demands that may hinder their engagement in meetings planned between regular business hours. Many of the ERIC strategies may not seamlessly map onto every context, nor will they capture every unique scenario pertaining to each unique knowledge user. As such, those engaging in the KM activity should be consulted on how best to tailor these strategies such that they can be applied effectively in each context. This can be done through discussion around scope of the strategy (e.g., does it capture each partner type appropriately, is the full scope of activities planned addressed) and appropriateness of the language (e.g., is the term “stakeholder” appropriate to utilize given its colonial origins? Is terminology like “incentive” relevant or is “compensation” more appropriate? ), among other factors that may be relevant in each unique context.

## Discussion

Engagement and partnership with knowledge users are critical to support KM practice; however, specific strategies are lacking to inform how they are implemented. While the evidence of patient partnership is emerging, practice is increasingly widespread and identification and application of relevant evidence-informed TMFs to this area adds credibility, transferability, actionability, and possibilities for future research. This commentary suggests that it is possible to apply existing implementation science TMFs to inform partnership approaches within KM as evidenced by the case example, which mapped strategies from the ERIC onto factors that support partner engagement in KM within the context of pediatric pain. The outcome of this work, which demonstrated the ability to map ERIC strategies onto KM practice and partnership factors is important for two reasons. First, it presents a tangible opportunity to link implementation science to practice, offering part of a solution toward the goal of closing the knowledge to action gap that exists in this area. Indeed, given the connection between the ERIC and the factors that support partnership from the earlier work, there is evidence to suggest that there are partnership aspects that appear to already exist within these strategies. Previous work by Perry and colleagues mapped ERIC strategies to their implementation project in primary care and same as this work, found that developing stakeholder interrelationships and train and educate stakeholders were the most commonly occurring clusters [26]. This not only mirrors the present findings but more importantly, suggests that these may be key ERIC clusters to look to for support with partnerships in implementation contexts. Practically, this highlights an opportunity for those looking to engage in partnership activities to consider how TMFs or taxonomies with relevant strategies can be used to inform how these relationships are developed and utilized. The second reason is that the opportunity to map strategies onto KM and partnership priorities speaks to a broader idea that the field of implementation science does not need to “reinvent the wheel” when it comes to developing strategies for implementing partnerships for KM initiatives; rather, there is preliminary evidence that existing implementation science TMFs may be applied to support these initiatives. Where TMFs and their related strategies align with and address core partnership considerations, such as meaningful engagement that sustains implementation, ongoing communication, and relationship building with diverse partners [18], leaders of KM initiatives will be better supported in their partner engagement. As this work suggests, there are in fact potentially multiple strategies that may serve to support a single partnership factor, introducing a flexible and comprehensive approach to how partnership strategies are selected. With that said, every TMF or strategy available may not seamlessly map onto each context. As such, care must be taken by those leading KM initiatives and partnerships to consult with partners and ensure strategies are tailored appropriately, much like how one would tailor an implementation strategy to a given context.

With these opportunities in mind, there are future development opportunities that emerge. The present commentary discussed one specific taxonomy that may be used to inform partnership implementation strategies. As has been discussed, however, there are other TMFs available that may also bear relevance to partnership. As such, a next step for implementation scientists is to review and identify other implementation science TMFs that may inform partnership activities. Moreover, when identifying relevant TMFs, it is worthwhile to consider language changes to these strategies to lend them to implementation of partnership, something that may also address present barriers to using TMFs for this purpose. Indeed, efforts are being made to suggest wording changes to the ERIC strategies to be more inclusive of the range of partners who may participate, and to make the strategies more practical [26]. For example, this could involve including language that specifies a broader set of partners and language that provides more specific steps about how a strategy would be enacted [26]. Based on the case example presented in this work, potential adjustments could include revising the strategy to “involve patients/consumers and family members” or to “involve patients/consumers and family members through structured opportunities to contribute to meetings.” Modifications such as these would not only be more inclusive, but would specifically highlight the approach to how this strategy can inform partnership. This will not only create a more comprehensive set of strategies to engage in partnership, but can facilitate the opportunity to select TMFs that may be more relevant to the context or KM activity at hand. It is indeed a shortcoming of the ERIC taxonomy more specifically that the strategies require contextualization and to be broken down in more detail to make them applicable. Language changes to the ERIC to inform partnership activities could inform more tangible applications of this taxonomy. Relatedly, there may also be opportunities to explore the use of other TMFs and situate the roles of partners within them. For example, the Interactive Systems Framework is designed to support partners in identifying their roles within implementation activities [27]. Such a framework could support partners in identifying opportunities to use strategies to engage and support implementation. Another future step is to address an underlying barriers to using TMFs in the first place, which is the inaccessibility of implementation science TMFs to individuals outside of the implementation science field. Efforts to make these TMFs and relevant strategies more accessible and usable to knowledge users (especially patient partners) and knowledge producers who are not familiar with implementation science is essential to support evidence-informed partnership approaches wherever they are practiced. Resources to simplify the purpose of strategies and present them in a plain language and easily implementable fashion (e.g., toolkits, education resources, checklists, interviews) are potential avenues through which the evidence on partnership could be translated and made more accessible to knowledge users and producers beyond the field of implementation science.

## Conclusion

Overall, there is opportunity to use existing implementation science TMFs to inform partnership approaches, especially when they are utilized in the context of KM activities specifically. Existing strategies may be especially impactful and supportive when they are selected and tailored to contexts alongside the partners within them. The application of existing implementation science TMFs to partnership represents a meaningful step toward filling the gap between the evidence that points to the importance of partnership and the steps toward engaging in practicing partnership in an evidence-informed manner.

## Data Availability

No datasets were generated or analysed during the current study.

## Abbreviations

CFIR: Consolidated Framework for Implementation Research
ERIC: Expert Recommendations for Implementing Change
IKT: Integrated Knowledge Translation
KM: Knowledge mobilization
TMF: Theories, models, and frameworks
